# Matrix type influences embedded patient-derived osteosarcoma organoid invasion and response to treatment

**DOI:** 10.3389/fphar.2026.1831729

**Published:** 2026-06-02

**Authors:** Luke Hipwood, Diana M. Vargas Reyes, Erik W. Thompson, Philip D. Rowell, Martin Lowe, Wayne Nicholls, Sugandha Bhatia, Dietmar W. Hutmacher, Christoph Meinert, Jacqui A. McGovern

**Affiliations:** 1 Faculty of Health, School of Biomedical Sciences, Queensland University of Technology, Brisbane, QLD, Australia; 2 Centre for Biomedical Technologies, Queensland University of Technology, Brisbane, QLD, Australia; 3 Gelomics Pty Ltd., Brisbane, QLD, Australia; 4 Translational Research Institute (TRI), Brisbane, QLD, Australia; 5 Max Planck Queensland Centre (MPQC) for the Materials Science of Extracellular Matrices, Queensland University of Technology, Brisbane, Australia; 6 The University of Queensland, Brisbane, QLD, Australia; 7 Centre for Genomics and Personalised Health, QUT, Brisbane, QLD, Australia; 8 Orthopaedics Department, Princess Alexandra Hospital, Metro South Hospital and Health Services, Brisbane, QLD, Australia; 9 Oncology Services Group, Children’s Health Queensland Hospital and Health Servies, Queensland Children’s Hospital, Brisbane, QLD, Australia

**Keywords:** 3D cell culture, biomaterial, decellularized extracellular matrix, hydrogel, lung, organoid, osteosarcoma

## Abstract

Osteosarcoma remains a lethal paediatric malignancy when it metastasises to lung, yet therapeutic progress has stalled for decades, in part because preclinical models poorly capture patient specific tumor microenvironments. Here, patient- derived osteosarcoma (PDOS) cells are assembled into uniform, matrix-free organoids and then embedded into photocrosslinkable extracellular matrices with independently tuneable stiffness and composition: collagen-rich gelatin methacryloyl (GelMA) or basement membrane protein-enriched lung decellularized extracellular matrix (dECM) methacryloyl (LungMA), each tuneable to soft (approximately 1 kPa) or stiff (approximately 4 kPa) conditions. Embedding converts otherwise compact organoids into invasive, expanding structures and increases resistance to doxorubicin relative to both 2D cultures and matrix-free organoids. Matrix identity influenced early invasion kinetics and treatment response, with LungMA promoting more aggressive invasion shortly after embedding and greater chemoresistance than GelMA, particularly under stiffer conditions. Image-based segmentation of core and invasive compartments revealed a critical divergence between metabolic viability readouts and functional invasion inhibition under chemotherapy, exposing limitations of conventional screening endpoints. These findings establish stiffness-controlled, tissue-derived extracellular matrix (ECM) organoid systems as a tuneable platform to interrogate microenvironment-driven osteosarcoma aggressiveness and to advance patient-specific assessment of therapeutic vulnerability in metastatic niche-like contexts.

## 1 Introduction

Osteosarcoma (OS), characterized by presence of immature osteoid, is the most common malignant bone tumor in children and adolescents. (Yenwongfai et al., 2022; Rothzerg et al., 2023; Yang et al., 2017). The five-year survival rate of patients with localized disease ranges between 60%–80%, whereas those with lung metastatic osteosarcoma have a poor prognosis with survival rates of 30% or lower. (Sadykova et al., 2020; Huang et al., 2019; Harris and Hawkins, 2022). Standard clinical treatment of OS typically involves limb-sparing surgery and multi-agent chemotherapy consisting of high-dose methotrexate, doxorubicin (DOX), and cisplatin (MAP) (Marina et al., 2016; Smrke et al., 2021; Ferrari and Serra, 2015). Despite improving patient outcomes when first introduced, survival outcomes for OS patients have stagnated for more than 4 decades, highlighting the urgent need for new therapeutic approaches and advanced pre-clinical models for understanding drug resistance and OS behavior in both primary OS and lung metastatic OS.

Pre-clinical OS research has traditionally relied on two-dimensional (2D) *in vitro* cancer cell cultures and *in vivo* animal models. However, 2D cell cultures do not accurately represent the complex three-dimensional (3D) tumor in its native microenvironment, exhibited through the lack of features such as cell-matrix-interactions, stiffness-dependent biophysical cues from the ECM, necrotic core formation, nutrient gradients, and dynamic matrix remodeling (Pignochino et al., 2009; Bruserud et al., 2005; Human Osteosarcoma Cell Lines Are, 1994; Hingorani et al., 2009; Lisle et al., 2008; Rodrigues et al., 2022). Furthermore, *in vivo* models possess biological, ethical, and practical constraints, such as differences in gene expression and immune response to humans, strict regulatory oversights, high costs, and long experimental timelines, making them less accessible and biologically relevant than 3D models (Negi et al., 2023). To address these challenges, researchers have adopted 3D matrix-free self-assembled cancer spheroids to study tumor formation and to evaluate potential therapeutic candidates for osteosarcoma (Monteiro et al., 2021; Sagheb et al., 2024; Pavlou et al., 2019; Bhatia et al., 2026). These spheroids allow better mimicking of tumor morphology, nutrient gradients, and chemotherapeutic diffusion, resulting in altered chemoresistance profiles compared to 2D. To better recapitulate tumor-extracellular matrix (ECM) interactions, recent studies have employed spheroid hydrogel embedding approaches that enable the evaluation of matrix dependent growth dynamics and chemoresistance (Monteiro et al., 2021; Sagheb et al., 2024; Naber et al., 2011; Akeda et al., 2009).

Pre-formed OS spheroids comprised of commonly used OS cell lines such as MG-63, U2OS, SAOS-2, and patient-derived immortalized cell lines (PDCLs) have been utilized for various purposes, such as the evaluation of OS in co-culture with human bone marrow-derived mesenchymal stem cells and osteoblasts to more faithfully recreate the diverse cell population of the bone, derivation of the relationship between *in vitro* cell proteomic data and OS chemoresistance in multiple cell lines, and recapitulation of the primary OS microenvironment through co-culture of PDCLs with M2 macrophages (Monteiro et al., 2021; Pavlou et al., 2019; Bhatia et al., 2026; Akeda et al., 2009; Arai et al., 2013; Pierrevelcin et al., 2022). Interactions between OS cells and the ECM vary markedly between patients in both primary tumors and lung metastases, which may contribute to the limited improvement in clinical outcomes over time (Wang et al., 2019; Zhou et al., 2020; Scott et al., 2011) Despite multiple reports of spheroid formation using OS cell lines, there is a significant lack of studies that apply pre-formed spheroid formation on non-immortalized patient-derived OS (PDOS) samples (hereafter termed organoids). To this extent utilizing 3D matrices for the investigation of PDOS organoids may be able to provide insight into patient-specific tumoral growth, invasion, and chemoresistance.

Basement membrane extracts such as Matrigel® are widely used as ECM surrogates in 3D patient-derived organoid workflows, largely due to their high laminin content which supports cell adhesion, survival, and organoid formation across multiple tissue types. However, these matrices exhibit unphysiological and poorly defined stiffness, typically in the range of 0.01–1 kPa, with substantial batch to batch variability (Soofi et al., 2009; Aisenbrey and Murphy, 2020; Slater and Partridge, 2017; Hughes et al., 2010). Their undefined composition, intrinsic growth factor content, and limited mechanical tunability restrict experimental control and confound interpretation of matrix specific effects. In addition, handling requirements such as cold chain processing, temperature sensitive gelation, and viscous workflow constraints contribute to reduced reproducibility and operational complexity (Soofi et al., 2009; Aisenbrey and Murphy, 2020; Slater and Partridge, 2017; Hughes et al., 2010; Hipwood et al., 2026). Collectively, these limitations make basement membrane extracts suboptimal for PDOS organoid embedding and invasion studies, where independent modulation of matrix stiffness and rigorous control of microenvironmental parameters are critical for dissecting tumor mechanobiology and therapeutic response.

In contrast, methacrylated gelatin (GelMA) is a well-characterized photocrosslinkable biomaterial widely used in 3D cell culture. Its mechanical properties can be precisely tuned by adjusting polymer concentration and light mediated crosslinking parameters, enabling controlled modulation of matrix stiffness, thereby supporting stiffness-defined three dimensional culture systems that recreate native human tissue stiffnesses (Bessot et al., 2023). Despite these advantages, GelMA is derived from gelatin and is therefore predominantly collagen-based, lacking key basement membrane associated ECM components such as laminins that are present in Matrigel and are often important for efficient patient-derived organoid establishment (Rezakhani et al., 2021; Gjorevski et al., 2016). Recently, we reported the development of LungMA, a methacrylated, decellularized and solubilized lung ECM that forms a hydrogel upon light exposure in the presence of a photoinitiator, while retaining a diverse and tissue-specific ECM protein profile enriched in basement membrane components (Hipwood et al., 2026). This diverse ECM composition may aid organoid establishment and development, or result in matrix-specific cell characteristics, as seen in the aforementioned study, where both matrix composition and stiffness impacted growth and response to DOX and cisplatin chemotherapy of encapsulated non-small cell lung cancer cells.

Additionally, our group recently characterized the molecular, phenotypic and functional attributes of OS cell lines and PDOS cultured in 2D, matrix-free/ULA, and in 3D Matrigel culture, with observations that gene expression and response to MAP chemotherapy were significantly different across culture platforms (Bhatia et al., 2026). These results suggested that the generation of 3D models represents a significant advancement in *in vitro* OS modelling compared to 2D methods; however, assessment of the impact of organoid behavior within matrix-type, matrix-stiffness, and cell-ECM invasion is warranted.

Therefore, to practically assess the behavior of PDOS within the context of basement membrane or gelatin-based microenvironments, we established and implemented the 3D-organoid embedding technique though ULA-based organoid formation and embedding in LungMA and GelMA at different stiffnesses. Prepared under soft (1 kPa) and stiff (4 kPa) conditions, LungMA and GelMA hydrogels were employed to examine the influence of the ECM composition and matrix stiffness on OS growth and response to treatment. PDOS organoids were embedded on day 3 following pre-formed organoid assembly and cultured in the hydrogels for an additional 14 days to determine their matrix-specific growth characteristics. ImageJ analysis was conducted to determine organoid growth and invasion into matrices over time. DOX, a first-line chemotherapeutic agent for treatment of OS, was used to assess the chemosensitivity of organoids under the selected matrix conditions. After DOX treatments, embedded and matrix free organoids were assessed for their metabolic activity, organoid size, morphology, and cellular invasion in LungMA and GelMA. We hypothesized that ECM-embedded organoids would be more chemoresistant compared to matrix-free conditions due to the activation of matrix-specific cell survival pathways, and that the basement membrane protein-rich microenvironment provided by LungMA would further increase chemoresistance and cell invasion compared to GelMA.

To our knowledge, this is the first study to apply ULA-based organoid formation on non-immortalized PDOS cells in a lung-based 3D *in vitro* model. Through the characterization of matrix-specific PDOS organoid growth and invasion, we have demonstrated the potential of stiffness-tunable LungMA and GelMA hydrogels in the context of *in vitro*, patient-specific pre-clinical evaluation of OS.

## 2 Materials and methods

### 2.1 Cell culture

The use of, and experimentation on, PDOS samples was approved by the Metro North Human Research Ethics Committee (HREC/2020/QRBW/61294) and the QUT Human Research Ethics Committee (approval no. 4550 and 3179), respectively. All work involving human-derived material was conducted according to the National Statement on Ethical Conduct in Research involving Humans (NHMRC) and in accordance with the standards and protocols of the Centre for Personalized Analysis of Cancers (CPAC, Brisbane, Australia). OS cells were isolated from a male with tibial osteosarcoma during surgical resection, who underwent MAP chemotherapy treatment with no reported issues. The PDOS cells were isolated using explant culture as previously described. PDOS cells were cultured in Advanced Dulbecco’s Modified Eagle’s Medium/Nutrient Mixture F-12 with L-glutamine (ADMEM/F-12) (Thermo Fisher, Lot# 3136899), 10% (v/v) fetal bovine serum (FBS) (Thermo Fisher, Lot# 2649790RP) and 1% (v/v) penicillin/streptomycin (P/S) (Thermo Fisher, Lot# 247746) (Bhatia et al., 2026). All cells were maintained in a cell culture incubator at 37 °C, 5% CO_2_, and 95% humidity. Cells were passaged at 85% confluency using 1 mL 0.25% (v/v) trypsin-ethylenediaminetetraacetic acid (EDTA) (Thermo Fisher, Lot# 3180743) per 75 cm^2^ cell culture flask with 2–3 min incubation at 37 °C or until cell detachment. Once detached, trypsin-EDTA was neutralized using respective growth mediums, then collected and pelleted via centrifugation for 5 min at 1,000 × *g*. The number of viable cells were then determined using an automated cell counter.

### 2.2 Organoid generation

**FIGURE 1:**
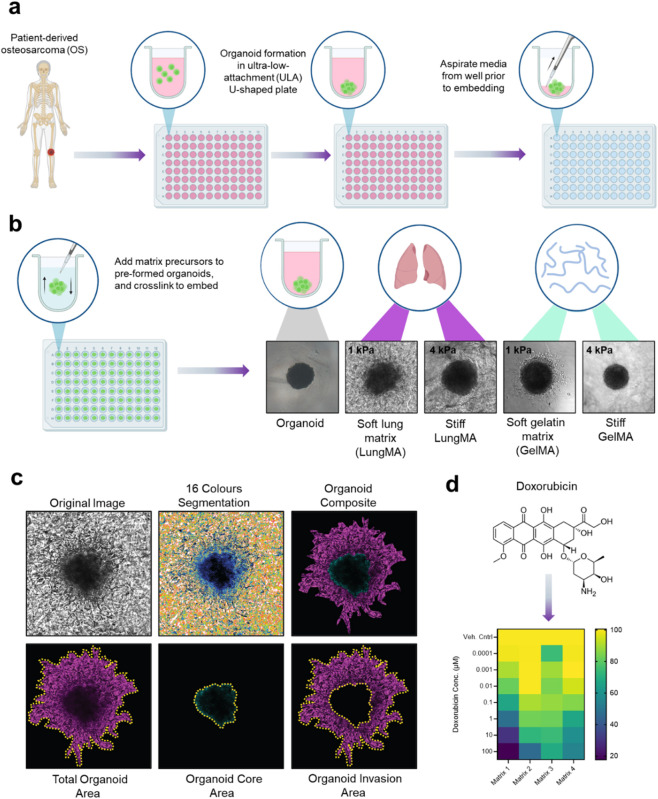
Workflow for patient-derived osteosarcoma (PDOS) organoid generation, embedding in photocrosslinkable ECMs, and analysis. **(a)** Method for generation of PDOS organoids. **(b)** On day 3 of culture, PDOS organoids were embedded in photocrosslinkable matrix precursors and crosslinked using visible light to prepare OS-matrix microenvironments. **(c)** Method for determination of organoid core and invasion area using brightfield images and ImageJ analysis. **(d)** Matrix-based response to doxorubicin (DOX) was used to evaluate the effect of matrix type and stiffness on OS response to chemotherapy.

Matrix-free, self-assembled organoids were formed using 96-well U-shaped bottom ultra-low attachment (ULA) plates (Corning, Cat. # 7007). Briefly, 4,000 PDOS cells were seeded per well and plates were centrifuged at 1,000 × *g* for 1 min to promote cell aggregation. Once centrifuged, 100 µL of growth media (ADMEM-F12 + 10% FBS +1% P/S) was added per well. An overview of this process is displayed in Figure 1a. Media (50% of total volume) was exchanged every 3 days, and organoid growth was monitored over the course of 14 days. Brightfield images were taken on days 1, 3, 5, 7,14 and 17 using an Olympus CKX41 inverted phase contrast microscope to observe organoid size and morphology over time.

### 2.3 Proteomic analysis of ECM derivatives used for preparation of hydrogel precursors

Proteomic analyses of lung dECM, gelatin and GelMA were conducted as previously reported (Hipwood et al., 2026). Once identified, the ECM protein profiles of samples were normalized to total ECM to determine the relative abundance of proteins within the total ECM.

### 2.4 Hydrogel precursor preparation

Two ECM-based hydrogel conditions were used for the encapsulation of spheroids, (i) GelMA derived from porcine skin (Gelomics, Lot# KL24J35); and (ii) LungMA, derived from porcine lung tissue, and developed in-house through decellularization and methacrylation as previously described (Hipwood et al., 2026). GelMA precursor solutions contained 5% (wt/v) GelMA (porcine skin, Type A), and 0.15% (wt/v) lithium phenyl-2,4,6-trimethylbenzoylphosphinate (LAP) (Gelomics, Lot# LS23A12) dissolved in phosphate-buffered saline (PBS) (Thermo Fisher, Lot# 3175885). LungMA precursor solutions were comprised of 1% (wt/v) LungMA and 0.15% (wt/v) LAP dissolved in PBS.

### 2.5 Determination of the degree of functionalization of hydrogel precursors

The degree of functionalization (DoF) of LungMA and GelMA was quantified using 2,4,6-trinitrobenzenesulfonate (TNBS) assay as previously described (Loessner et al., 2016). In brief, pepsin-digested lung tissue and dECM homogenates were dissolved in 0.1 M NaHCO_3_ buffer to prepare a dilution series ranging from 250 μg/mL to 0 μg/mL. Triplicates of 200 μL of lung dECM, LungMA, gelatin, and GelMA solutions were transferred to a 96-well plate, mixed with 100 μL of 0.01% (wt/v) TNBS solution, and incubated for 2 h protected from light. The plate absorbance was measured at 335 nm. The DoF was determined as the percentage of amine reduction in methacrylated samples compared to non-methacrylated samples.

### 2.6 Compression testing of hydrogels

Hydrogels were allowed to swell to equilibrium overnight in PBS at 37 °C. Following swelling, hydrogels were imaged using a Nikon SMZ25 stereomicroscope at ×0.46 magnification, and surface area of the hydrogels was determined using ImageJ (version 1.54r) software. The hydrogels were then submerged in a 37 °C PBS-filled water bath and compressed in an unconfined configuration using an Instron 5848 Microtester (Instron®, Norwood MA, USA) and non-porous aluminium indenter, at a strain rate of 0.01 mm/s. The Young’s moduli (E) of the hydrogels were determined as the slope of stress-strain curves at 10%–15% strain (Loessner et al., 2016).

### 2.7 Organoid embedding

Organoids pre-formed in ULA plates were embedded in LungMA and GelMA ECMs, respectively, on day 3 of culture. Prior to encapsulation, growth medium was completely aspirated from each organoid-containing well. Then, 30 µL of the respective hydrogel precursor solutions were added to each well. The ULA plates containing the hydrogel-organoid mixtures were then centrifuged at 1,000 × *g* for 1 min to ensure organoids were located in the center of each well. The plates were then photocrosslinked using a 405 nm light (approx. 12 mW/cm^2^) in a LunaCrosslinker™ (Gelomics) for 15s (soft LungMA), 1 min (soft GelMA), and 4 min (stiff GelMA and stiff LungMA), respectively. After crosslinking, 100 µL of fresh growth media was added to each sample, and plates were further incubated in a cell culture incubator as previously described.

### 2.8 Brightfield organoid area and invasion analysis

All image analysis was conducted using ImageJ according to a standardized workflow. Brightfield images acquired at 4× or ×10 magnification using an Olympus CKX41 inverted phase contrast microscope were converted to 8-bit greyscale prior to analysis. Where required, a 16-color lookup table was applied to assist visual discrimination of invasive regions. Brightness and contrast were adjusted uniformly to optimize spheroid and invasive cell visibility. The total organoid area, defined as the organoid core plus the invasive outgrowth, was delineated using the freehand selection tool. This region of interest was duplicated, and the organoid core was isolated by thresholding and converted into a binary mask. Core area and circularity were quantified using the Analyze Particles function. The invasive area was calculated by subtracting the organoid core area from the total organoid area. A representative macro illustrating the analysis pipeline is provided in Supplementary Figure S3.

### 2.9 Live/dead viability staining of organoids

The viability of matrix-free organoids on day 7 and 14 of culture, and embedded organoids at the endpoint of DOX treatment (day 4 embedding), was determined using a fluorescein diacetate (FDA) (Thermo Fisher)/propidium iodide (PI) (Thermo Fisher) cell viability assay. In brief, cell culture media was aspirated from samples, which were washed thrice with 100 µL of 1 × PBS. Then, PBS was aspirated, and 100 µL of viability stain solution containing 10 μg/mL FDA and 5 μg/mL PI in 1 × PBS were added to each sample and incubated at room temperature for 5 min. Samples were washed thrice with 100 µL of PBS and imaged using a FLUOVIEW™ FV4000 confocal microscope. Z-stacks were captured at ×10 magnification with 1 µm slice intervals, and maximum intensity projections were used for live/dead image analysis, whereby the number of cells present in green (live) and red (dead) channels were used to determine overall viability.

### 2.10 Immunofluorescence organoid staining

On day 17 of matrix-free culture and day 14 of embedded culture, samples were fixed in 100 µL 4% paraformaldehyde (PFA) for 1 h at RT, then washed thrice with 100 µL 1 × PBS. Post-fixation, samples were permeabilized using 100 µL 1% (v/v) Triton X-100 (Merck Millipore, CAS# 9036-19–5) in 1 × PBS for 20 min at 4 °C on an orbital shaker. Then, permeabilizing solutions were removed, and samples were blocked using 100 µL 5% (wt/v) bovine serum albumin (BSA) (Thermo Fisher Scientific) at 4 °C on an orbital shaker for 2 h. Samples were then washed thrice with 1 × PBS. Anti-vimentin primary rabbit (Rb) monoclonal antibody (mAb) (abcam, cat. Ab92547, Lot# GR325879) (stock concentration 0.25 mg/mL) solution was prepared through the addition of primary vimentin mAb to 1% (wt/v) BSA at a 1:400 dilution. Subsequently, 100 µL of the antibody solution was added to each sample, followed by overnight incubation at 4 °C on an orbital shaker. Solutions were aspirated and replaced with 1% (wt/v) BSA and incubated at 4 °C on a plate shaker for 2 h. This step was repeated thrice. Samples were then incubated with 100 µL of goat anti-Rb, Alexa Fluor 647-conjugated secondary antibody (abcam, Lot# AB150110) solution (1:200 dilution in 1% (wt/v) BSA) (stock concentration 2 mg/mL) at 4 °C on an orbital shaker for 45 min. Afterwards, 100 µL of 1:200 dilution, Alexa Fluor 488-conjugated phalloidin (abcam, Lot# A123879) (stock concentration 0.5 mg/mL) in 1% (wt/v) BSA was then added to each sample and incubated for 1 h. A 4′,6-diamidino-2-phenylindole (DAPI) (Sigma Aldrich) cell nuclei stain prepared at 1:1000 dilution in 1% (wt/v) BSA (stock concentration 5 mg/mL) was then added to each sample in 100 µL aliquots. Samples were then incubated at 4 °C on an orbital shaker overnight. Once staining was complete, all staining solutions were removed, and samples were washed thrice with 1% (wt/v) BSA for 6-h intervals prior to confocal imaging.

To characterize the morphological characteristics of embedded PDOS organoids, a minimum of 100 regions of interest (ROIs) were captured per sample at ×10 magnification using a FLUOVIEW™ FV4000 confocal microscope and stitched together to create composite organoid invasion images. Once stitched, the classification of ROIs were determined using ROI segmentation and ROI selection, to denote core, intermediate invasive, and distally invasive regions (Supplementary Figure S1). Core regions were identified as regions with highly dense, circular cell morphologies, with organoid-like borders, like the cores observed through brightfield microscopy in both matrix-free and matrix-embedded conditions. Intermediate invasive regions were identified as regions directly neighboring (0–100 µm distance) from the selected core regions. Distally invasive regions were identified as the remaining regions outside of the core and intermediate invasive zones.

### 2.11 Chemotherapeutic treatment

Matrix-free (2D and matrix-free organoid) and embedded PDOS were subjected to chemotherapeutic treatment from day 3 (the day of embedding in lungMA or GelMA ECMs) to day 7 (day 4 after embedding) of culture. PDOS cultures were treated with either vehicle control (dimethyl sulfoxide (DMSO)) (Sigma Aldrich, Lot# RNBK6387) in growth medium, or one of 7 respective dose concentrations of doxorubicin hydrochloride (DOX) solubilized in DMSO, ranging from 1 × 10^−4^ μM–100 µM DOX. To administer agents, 50 µL of growth media was exchanged for either DMSO or DOX on days 3 and 5 of culture. The rationale for conducting DOX treatment immediately after embedding was that doing so would enable the observation of any invasion inhibition, as by day 4 of embedding (day 7 total time), cells from the embedded organoids were already invading outside of the core into the surrounding matrix in all embedded samples.

### 2.12 Metabolic activity assay

The metabolic activity of cell cultures was determined using a PrestoBlue® metabolic activity assay (Thermo Fisher Scientific) following the manufacturer’s instructions. Briefly, samples were washed thrice with 100 µL 1 × PBS. Then, 100 µL of working PrestoBlue® solution comprised of 90% growth medium and 10% PrestoBlue® stock solution. Then, PrestoBlue®-laden wells were incubated in a cell culture incubator following previously described incubation conditions for 2 h. Following incubation, the fluorescence of each sample was read at an excitation wavelength of 540 nm and emission wavelength of 590 nm using a CLARIOstar Plus spectrophotometer (BMG Labtech).

### 2.13 Statistical analysis

All statistical analyses were performed using GraphPad Prism v10. Technical replicates (n) refer to individual samples, and biological replicates (N) refer to independent experiments performed on separate days. Unless otherwise specified, data are presented as mean ± standard deviation. Heatmap scores represent mean values.

## 3 Results and discussion

Advancements and utilization of three-dimensional (3D) culture platforms in the form of ULA-based spheroid and organoid generation offers physiologically relevant and biologically accurate models for investigating OS pathophysiology and preclinical therapeutic screening. Both scaffold-free and scaffold-based spheroid/organoid models recapitulate the tumoral characteristics, such as tumor-like aggregation, nutrient diffusion and hypoxic gradients and scaffold-based models recapitulate the tumour-ECM dynamics which better mimics the tumor complexity and drug response profiles. In our recent assessment of PDOS organoids, where we compared their morphological, transcriptomic and chemoresistance behavior to OS cell lines, we observed distinct molecular and cellular profiling of the generated 2D and 3D OS models, showing that they were significantly more chemoresistant in 3D compared to 2D conditions (Bhatia et al., 2026). Therefore, in this study we utilized the ULA-based spheroid formation technique for generation of consistent and reproducibly-sized OS PDOs, then embedded them in basement membrane protein-rich LungMA hydrogels and collagen-rich GelMA hydrogels, respectively, with the aim of characterizing OS PDO behavior, invasion, and response to DOX therapy (Figure 1), thus establishing methods for *in vitro* 3D OS PDO assessment in different ECM microenvironments.

### 3.1 Characterization of matrices used for organoid embedding

**FIGURE 2:**
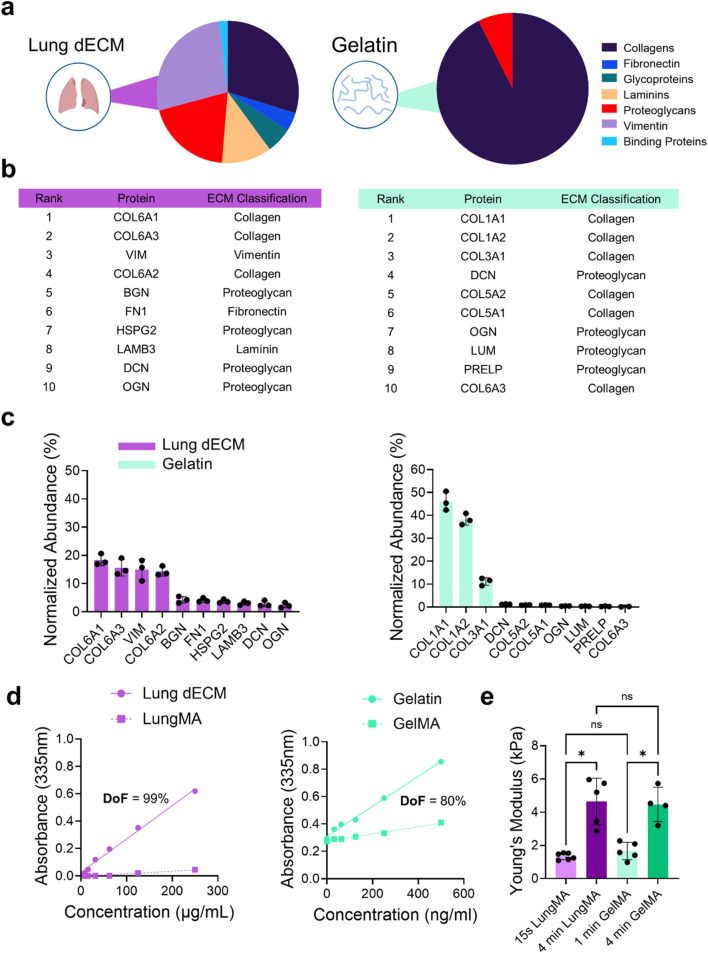
Characteristics of ECM-derivatives and their methacrylated counterparts used for PDOS organoid embedding. **(a)** Distribution of abundant matrisome protein classes present in porcine-derived lung dECM and gelatin determined through proteomic analyses. **(b)** Classification, rank, and **(c)** normalized abundance of the top 10 ECM proteins present in lung dECM and gelatin normalized to total ECM proteins. N = 1. n = 3. Data expressed as mean ± SEM. **(d)** Degree of functionalization (DoF) of methacrylated lung dECM (lungMA) and gelatin (GelMA), determined via amine quantification. N = 1. n = 6. **(e)** Young’s modulus (stiffness) (kPa) of photocrosslinked LungMA and GelMA hydrogels determined through compression testing. N = 1. n = 4-6. Data expressed as mean ± STDEV.

The preliminary assessment of the proteomic analyses was conducted from the biomaterials used to manufacture the hydrogel precursors: porcine-derived lung dECM and porcine-derived gelatin. We found that lung dECM was constituted of a variety of matrisomal ECM proteins, such as collagens, laminins, fibronectin, proteoglycans, and vimentin (Figures 2A–C), representative of the diverse composition of the basement membrane (Hoffman et al., 2023; Åhrman et al., 2018). Being a collagen derivative, we found that gelatin was majorly comprised of collagens type I and type II, with trace amounts of proteoglycans (Figures 2a–c). These findings are similar to previous reports and demonstrates the absence of laminins in gelatin in compared to lung dECM (Windarsih et al., 2025; Zhu et al., 2023; Ward et al., 2018). Gelatin’s composition makes it desirable for general cell culture, due to its high material reproducibility and controlled rheological properties; however, it lacks pivotal ECM cues for organoid establishment, specifically laminins, attributed towards the ability to successfully support organoid establishment (Rezakhani et al., 2021; Gjorevski et al., 2016). Lung dECM, on the other hand, contains laminins in addition to many other ECM proteins that may be more suitable for patient-derived OS isolation, expansion, and organoid culture (Figure 1).

After assessing the matrisome profiles of lung dECM and gelatin, both materials were methacrylated to generate photocrosslinkable hydrogel precursors, LungMA and GelMA. Methacrylation enables controlled covalent crosslinking under visible light, allowing modulation of bulk mechanical properties through defined crosslinking parameters. Stiffness is a major biophysical property of the extracellular matrix and is known to regulate cancer cell behavior through mechanotransduction pathways, including integrin-mediated cell–ECM adhesion and downstream signaling cascades (Pallarola et al., 2017; Rühl et al., 1999; Wang and Pan, 2020; Voiles et al., 2014; Liu et al., 2024; Wishart et al., 2020; Dekker et al., 2026; Weiss et al., 2023). Therefore, the ability to reproducibly tune stiffness represents a critical parameter in the design of 3D culture systems intended to interrogate tumor phenotypes.

Both lung dECM and gelatin contain lysine residues that remain accessible following solubilization and processing, enabling efficient methacrylation. As shown in Figure 2d, high degrees of functionalization were achieved (99% for LungMA and 80% for GelMA), confirming successful chemical modification. Following methacrylation, the Young’s modulus of the photocrosslinked hydrogels was determined under defined light exposure conditions. For LungMA, crosslinking for 15 s and 4 min produced hydrogels in the soft and stiff regimes, respectively. For GelMA, crosslinking for 1 min and 4 min generated comparable soft and stiff conditions. Based on compression testing (Figure 2e), we selected approximately 1 kPa as the soft condition and approximately 4 kPa as the stiff condition, as these stiffnesses generally reflect the healthy lung microenvironment stiffness (soft, 1 kPa), and fibrotic or tumor-like lung microenvironment (stiff, 4 kPa), respectively, whilst keeping in mind that tissue stiffness values are highly dependent on the method used, therefore rendering our selection more exploratory and representative than a direct reflection of *in vivo* tissue stiffness (Sicard et al., 2018; Sicard et al., 2017; Polio et al., 2018). Furthermore, the chosen photocrosslinking method of hydrogel preparation does not impact cell viability, as demonstrated by previous studies where 8 min and 15 min crosslinking times have been used to create stiff hydrogels without cytotoxic effects (Hipwood et al., 2026; Bock et al., 2023).

### 3.2 Matrix-free growth of PDOS organoids

**FIGURE 3:**
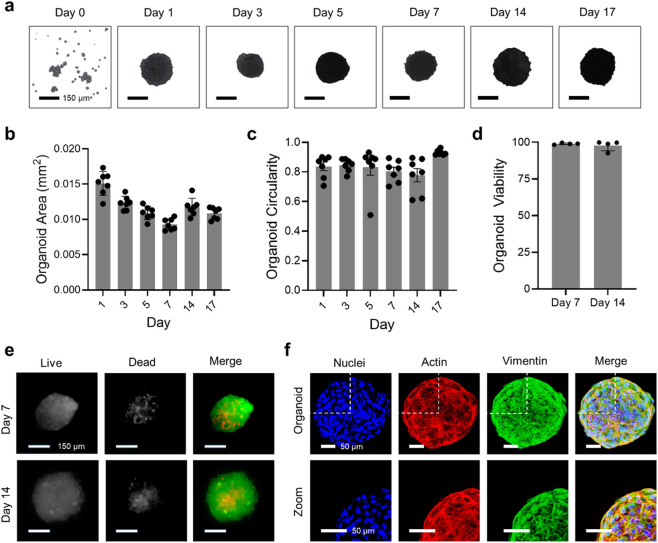
Matrix-free PDOS organoid morphology over time. **(a)** Brightfield images of self-assembled OS organoids over time. Scale bar = 150 μm. **(b)** Measurement of organoid area via ImageJ analysis. N = 2. n = 3-4. Error bars = STDEV. **(c)** Organoid circularity was measured via ImageJ. N = 2. n = 3-4. Error bars = STDEV. **(d)** PDOS organoid viability was measured through fluorescein diacetate (FDA) (live)/propidium iodide (PI) viability assay and ImageJ analysis. N = 2. n = 2. **(e)** Representative images of FDA/PI-stained PDOS organoids on day 7 and 14 of culture. Scale = 150 μm. **(f)** Fluorescent staining of cell nuclei (DAPI, blue) actin filaments (Alexa Fluor 488-conjugated Phalloidin, green) and vimentin (Alexa Fluor 647, red) on day 14 of culture reveal densely packed, circular morphology of matrix-free PDOS organoids. Scale bar = 50 μm.

Before evaluating how these compositionally distinct, stiffness-tunable matrices regulate PDOS behavior following embedding, we first established a reproducible baseline by generating uniform matrix free PDOS organoids suitable for controlled, like-for-like comparisons across embedding conditions (Figure 3). PDOS cells spontaneously formed organoids from multicellular aggregates on day 1 of culture (Figure 3a). These organoids decreased slightly in size between day 1 (0.015 mm^2^) and day 3 (0.01 mm^2^) until day 7, likely due to cellular condensation (Figure 3b) (Caprio and Burdick, 2022) The use of ULA plates for pre-formation of organoids resulted in reproducible organoid formation across independent experiments (CV = 9.5% for N = 2). PDOS organoid circularity remained consistent over time at ∼0.8, indicating uniform cell aggregation. These results suggested that day 3 would be the optimal timepoint for PDOS organoid embedding in ECM mimics, as delaying the embedding process for later timepoints would be redundant, as morphological changes beyond this culture period were immaterial.

Fluorescence imaging of live and dead cells showed that there were minimal dead cells on day 7 and day 14 of culture, but that on day 14, dead cells were localized to the organoid core (Figure 3E). As shown by Bhatia et al, the timepoint for necrotic core development can vary between OS cells in both PDOS and OS cell lines, as necrotic cores formed in one PDOS organoid on day 3, but not in a second, while the same trend was observed when comparing SAOS-2 spheroids (no necrotic core) and U2OS spheroids (necrotic core) (Bhatia et al., 2026). Our results indicate that for the patient-specific material used in our study, extended culture periods may induce the development of a necrotic core. Overall viability assessment indicated that the method used for generating both PDOS organoids was compatible for use in further experiments due to high organoid viability. Organoid morphology was made further evident through DAPI, conjugated antibody staining of cell F-actin (filamentous actin), and vimentin respectively, which revealed a compact spherical shape, with high nuclear density and vimentin expression, indicative of mesenchymal lineage (Figure 3F).

### 3.3 Growth and invasion of embedded PDOS organoids

**FIGURE 4:**
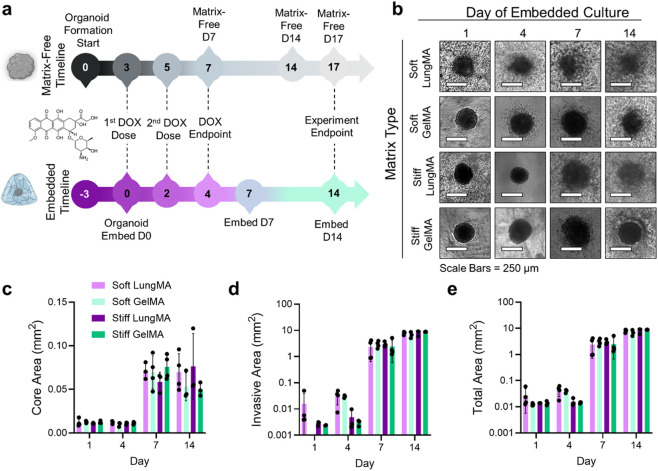
Morphology and invasion of ECM-embedded PDOS organoids over time. **(a)** Brightfield images of PDOS organoids embedded in soft and stiff LungMA and GelMA matrices, respectively, reveal increased cell migration from organoid core to surrounding matrix growth over time; **(b)** Timeline for PDOS organoid embedding. Organoids were embedded into matrices on day 3 of matrix-free culture. Scale bars = 250 μm. Organoid core **(c)**, invasive (d), and total **(e)** areas determined via ImageJ analysis. N = 1. n = 4. Error bars = STDEV.

Following matrix-free formation, organoids were embedded on day 3 into soft (1 kPa) or stiff (4 kPa) LungMA or GelMA hydrogels, respectively, and monitored over time (Figures 4a,b). Brightfield imaging and ImageJ-based segmentation enabled separate quantification of the core area and invasive (non-core) area over 14 days of embedded culture (Figures 4c–e). Across all embedded conditions, invasion increased markedly over time (Figure 4d). Invasive areas were low at day 1 and day 4 post-embedding, then increased substantially by day 7 and remained high at day 14 (Figure 4d). This trend is also reflected in total area measurements (core plus invasive area; Figure 4e). The core area increased from early timepoints (day 1 and day 4) to day 7 and then remained within a similar range through day 14 for all conditions (Figure 4c). Importantly, the core area values in Figure 4c are in the ∼0.01 mm^2^ range at day 1 and day 4, rising to ∼0.05–0.08 mm^2^ at day 7 and day 14 depending on condition.

While all experimental groups showed strong cell invasion by later timepoints, early behavior differed with stiffness and matrix type. At day 1 post-embedding, organoids in soft LungMA showed a larger invasive area than other groups, whereas soft GelMA and both stiff matrices displayed comparatively limited invasion (Figure 4d). By day 4, invasion increased in both soft matrices (soft LungMA and soft GelMA), while invasion in stiff matrices remained relatively constrained (Figure 4d). By day 7 and day 14, invasion areas converged across groups, with all conditions showing large areas of invasion (Figures 4d,e). Therefore, in this dataset, stiffness primarily influenced early invasion kinetics, with stiffer matrices delaying invasion during the first several days post-embedding (Figure 4d).

Mechanistically, this early stiffness dependence is consistent with the established influence of matrix mechanics on cell migration through mechanotransduction and integrin-mediated adhesion signalling (Gkretsi and Stylianopoulos, 2018; Wang et al., 2021; Zhang et al., 2025). Various ECM proteins within ECM can contribute towards this behavior. For example, collagen type VI, the most abundant protein in the lung dECM, is a loose pericellular microfibrillar network, that favors transient cell-ECM adhesion formation, quick adhesion release, and rapid matrix metalloproteinase (MMP)-based ECM degradation, while collagen type I, the most abundant protein found in gelatin, and the most abundant protein in bone, possesses the RGD (Arg-Gly-Asp) binding motif, that promotes relatively high cell-ECM adherence (Aumailley et al., 1989; Liu et al., 2024; Chen et al., 2013; Jonker et al., 2015; Pallarola et al., 2017). Here, matrix composition and stiffness influences early invasion kinetics; LungMA composition promotes earlier invasion under soft conditions, stiff matrices delay early invasion. However, the present study did not directly measure mechanotransduction pathways, focal adhesion dynamics, or matrix remodeling, so these mechanisms should be considered plausible contributors rather than demonstrated causes. Within the scope of the presented data, the robust conclusion is that embedding enables invasion that is quantifiable, and early invasion kinetics differ by stiffness and matrix type (Figure 4). The higher day 1 invasion observed in soft LungMA could be consistent with specific compositional cues (Figure 4d), but this study does not isolate specific ECM ligands or receptors, so the data supports an association rather than a causal assignment (Jonker et al., 2015; Pallarola et al., 2017).

After determining the morphological characteristics of matrix-free, self-assembled PDOS organoids, we embedded them on day 3 of culture (Figure 4a), the same timepoint for the first dose of DOX in treated cultures. (Figures 4b,d). The core areas of embedded organoids were comparable to non-embedded organoids on days 1 and 4 after ECM embedding, ranging between 0.01–0.0125 mm^2^ (Figure 4c). However, by day 7, embedded organoid core sizes increased to 0.02–0.08 mm^2^ for all matrices. The organoid core size was consistent for soft and stiff LungMA as similar core area was observed for day 14 embedded culture.

While the evaluated invasive areas of organoids showed significant increase by day 7 and day 14, irrespective of their stiffness and ECM composition, there was markedly reduction in cellular invasion of organoids within stiff ECM compositions at early time-points of day 1 and day 4 assessment (Figure 4d). Soft LungMA matrices supported higher proportion of cellular invasion on day 1 post-embedding (Figures 4b,d), while stiff LungMA and GelMA matrices showed restricted invasion at this timepoint. Interestingly, cell invasion was minimal to negligible in soft GelMA on day 1. By day 4 of culture, organoids embedded in soft LungMA and soft GelMA matrices increased invasive areas to ∼0.05 mm^2^, while organoids embedded in stiff matrices displayed similar invasive area coverage to day 1 post-embedding (Figure 4d). On day 7 of embedded culture, organoids embedded in all matrices displayed high ECM invasion, with invasion areas of 2.33 ± 1.69 mm^2^ in soft LungMA, 3.01 ± 0.71 mm^2^ in stiff LungMA, 3.06 ± 0.84 mm^2^ in soft GelMA, and 2.41 ± 1.83 mm^2^ in stiff GelMA. This increase in invasion was further observed on day 14 of embedded culture, where the invasive area of embedded organoids was ∼8 mm^2^ for all matrices.

These results indicate that the behavior of embedded organoids is regulated by both matrix composition and stiffness (Caprio and Burdick, 2022; Gkretsi and Stylianopoulos, 2018; Wang et al., 2021; Caprio and Burdick, 2022; Gkretsi and Stylianopoulos, 2018; Wang et al., 2021; Zhang et al., 2025; Aumailley et al., 1989; Liu et al., 2024; Chen et al., 2013; Jonker et al., 2015; Pallarola et al., 2017; Rühl et al., 1999).Overall, these results demonstrate that when embedded, organoid core and invasion areas both increase over time, with higher initial cellular invasion in soft LungMA compared to other matrices on day 1 post-embedding, and overall higher invasion indexes/factor in soft matrices compared to stiff matrices on day 4 of embedded culture. Organoids embedded in stiff LungMA displayed the highest invasion on day 7 of culture, however, stiffness and matrix-dependent invasion area was non-significant by day 14 of embedded culture.

**FIGURE 5:**
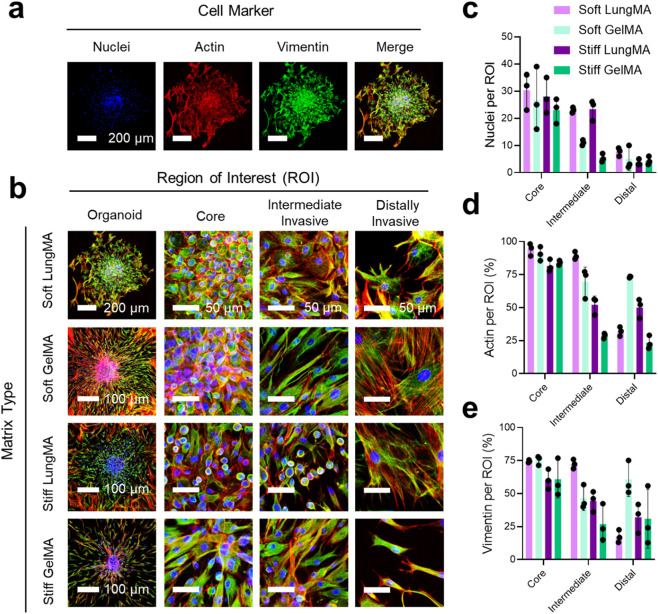
Fluorescence staining of PDOS organoids embedded in LungMA and GelMA matrices at day 14 of culture. **(a)** Cell marker panel used for identification of organoid cell structures. Nuclei (blue) indicate the number of cells present, while F-actin (red), a cytoskeletal marker, reveals cell morphology alongside vimentin, a mesenchymal-specific marker related to tumoral aggression; **(b)** Embedded organoid regions of interest (ROI) regarding core, intermediate invasive and distally invasive cultures. For each culture, ROIs were captured to form stitched whole organoid images. **(c)** The number of nuclei per ROI was determined through ImageJ analysis of nuclei channel (blue) of each ROI post-channel split. N = 1. n = 3. Error bars = STDEV. **(d)** F-actin (red) and **(e)** Vimentin (green) area per ROI (%) was determined as the area (%) within each ROI with actin or vimentin-positive fluorescent signal, respectively. N = 1. n = 3. Error bars = STDEV.

Despite similarities in invasion area by day 14 of embedded culture, PDOS organoids exhibited matrix-specific cell organization, density, and morphology at this timepoint, as seen in Figure 5. Fluorescently labelled nuclei, actin, and vimentin revealed the high cellular density of organoid cores, and cells invading to intermediate and distal areas from the core in all matrix types (Figures 5a,b). Quantification of the number of nuclei per ROI showed that soft and stiff LungMA groups supported the highest density in intermediate invasive areas, but there was no difference observed across matrix conditions when comparing cellular presence at distally invasive areas based on nuclei per ROI (Figure 5c).

Through quantification of actin and vimentin per ROI, we found that the soft matrices showed less constricted cytoskeletal arrangement as compared to stiff matrices in the embedded organoid cores. This was also the case in intermediate invasive areas regarding actin (Figure 5d); however, soft LungMA promoted higher vimentin coverage than all other matrix types in these areas (Figure 5e). In distally invasive ROIs, actin and vimentin were most highly expressed in soft GelMA and stiff LungMA-embedded organoids. Regarding cell morphology, distally invasive cells in stiff LungMA appeared more elongated and linearly organized than those in soft LungMA (Figure 5b). In GelMA matrices, increased stiffness appeared to reduce intermediate invasive and distally invasive cell size, as shown through actin coverage (Figure 5d). Furthermore, distally invasive cells in stiff LungMA and soft GelMA appeared more adherent, made apparent through their elongation and 2D-like morphology, than other matrices. These results further suggest that the PDOS organoids exhibit matrix-specific morphologies when embedded.

### 3.4 Organoid response to DOX treatment

**FIGURE 6:**
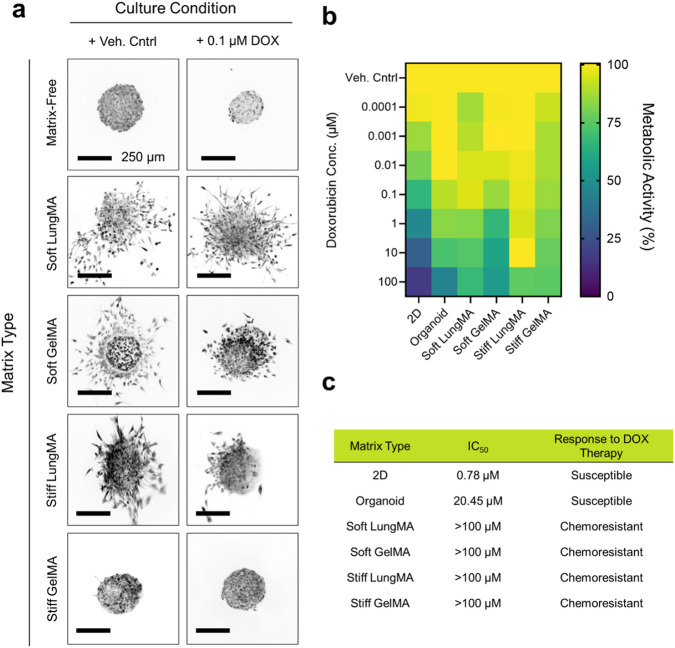
Doxorubicin (DOX) treatment of matrix-free and embedded PDOS organoids. **(a)** Fluorescein diacetate (FDA)-based staining of live PDOS organoids treated with mid-concentration DOX (0.1 µM) was conducted at endpoint of treatment (day 4 post-embedding). **(b)** Metabolic activity of matrix-free and embedded organoids determined on day 7 of culture (day 4 post-embedding). The relative metabolic activity of each sample was determined through normalization to respective group vehicle controls. Heatmap values represent mean. N = 1. n = 6. **(c)** IC_50_ values for PDOS cultures was determined via [inhibitor] vs. response variable slope (four parameters) non-linear regression.

**FIGURE 7:**
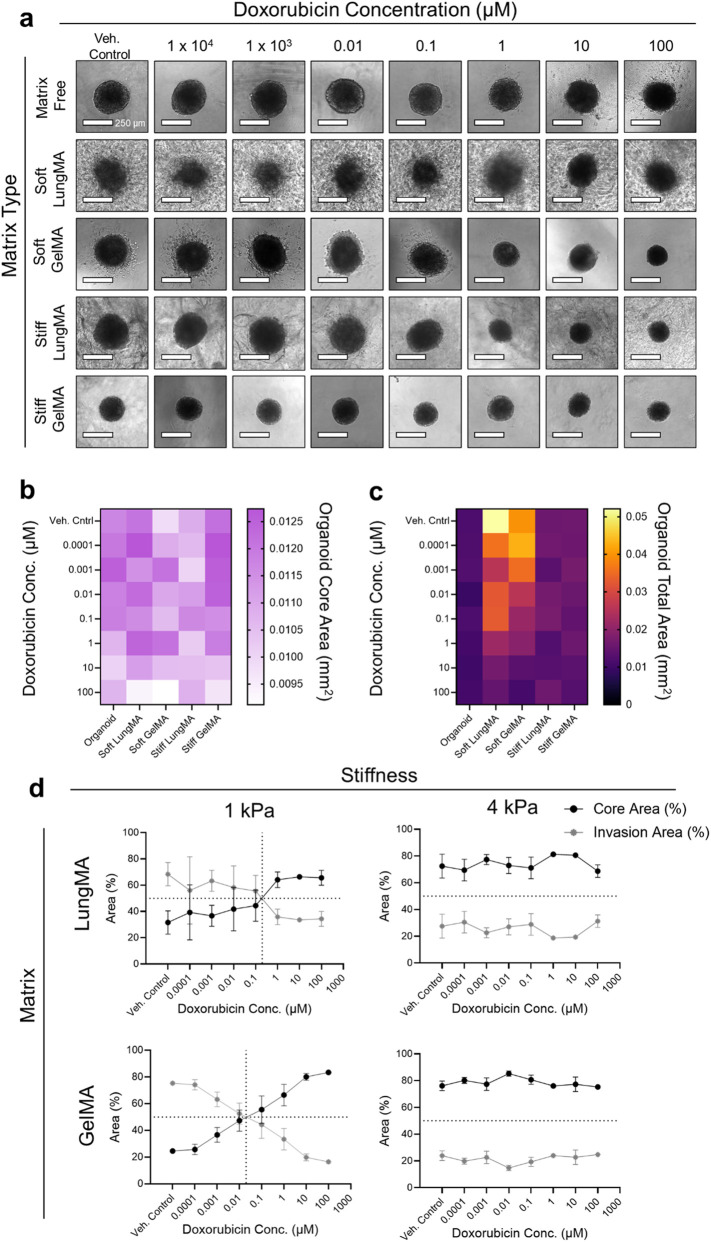
Morphological response of PDOS organoids to treatment with increasing DOX concentration. **(a)** Brightfield microscopy of PDOS organoids treated with DOX demonstrates that core and invasive areas were reduced in all groups with increasing DOX concentration. **(b)** Quantification of spheroid core areas revealed that core size was slightly decreased with increasing DOX concentration for most matrix types. N = 1. n = 6. Error bars = STDEV. **(c)** Total organoid area quantification shows that groups with high invasion, such as soft LungMA and soft GelMA, undergo a decrease in total cell area with increasing DOX concentration. N = 1. n = 6. Error bars = STDEV. **(d)** Quantification of the percentage of core and invasive cell areas in relation to total cell coverage acts as an indicator for drug-based invasion inhibition. N = 1. n = 6. Error bars = STDEV.

On day 3 and day 5 of culture (corresponding to day 0 and day 2 following embedding in ECMs), matrix-free and ECM-embedded PDOS organoids were treated with doses of DOX across a concentration range of 1 × 10^−4^ μM–100 µM (Figure 4a). The response to DOX treatment was evaluated using a PrestoBlue® metabolic activity assay (Figure 6b), and ImageJ analysis of organoid core and invasion areas on day 4 following embedding (Figure 7). This timeframe was selected because, as shown in Figure 5, invasive outgrowth became extensive at later stages of culture. At day 4 post-embedding, however, the relatively low degree of confluence enabled more accurate quantification of invasion inhibition compared with the highly confluent conditions observed at days 7 and 14.

Across all embedded conditions, increasing DOX concentration was associated with a reduction in both metabolic activity and invasive area (Figures 6, 7). Matrix-free organoids exhibited greater chemoresistance compared to 2D cultures, while matrix-embedded organoids demonstrated the highest level of chemoresistance among the tested culture systems. Notably, embedded organoids retained metabolic activities exceeding 50% even at the highest DOX concentration tested (100 µM), precluding reliable determination of IC_50_ values under these conditions (Figure 6b).

Potential concerns regarding limited drug penetration within hydrogel matrices may arise; however, the decrease in invasive cells is indicative of DOX penetrating through the respective matrices, in addition to the mesh sizes of the hydrogels accommodating the diffusion of DOX (Hipwood et al., 2026; Dekker et al., 2026). Future studies may enable more accurate determination of the IC_50_ by increasing chemotherapeutic concentrations, volumes, number of doses, or treatment duration. The parameters for treatment chosen for this study were based on 2D and non-embedded PDOs organoid results; however, our results show that future embedded organoid treatment approaches should be further adapted and optimized.

Live cell staining of DOX treated embedded samples revealed that following exposure to 0.1 µM DOX, embedded organoids remained viable, although matrix-specific reductions in invasive cell populations were observed relative to vehicle control groups (Figures 6a, 7). Metabolic chemoresistance was influenced by both matrix composition and stiffness, with PDOS organoids in lung-based microenvironments exhibiting increased chemoresistance compared to gelatin-based microenvironments. From the matrix conditions tested, PDOS organoids cultured in stiff LungMA demonstrated the greatest metabolic chemoresistance, suggesting that the combined biochemical composition and mechanical stiffness of the LungMA matrix promotes a more resilient tumor phenotype under these microenvironmental conditions.

In addition to decreasing metabolic activity, increasing DOX concentration was also associated with a reduction in both organoid core (Figure 7b) and the total area (Figure 7c). As shown in Figure 7c, soft matrices promoted the highest total organoid area, with vehicle control PDOS organoids cultured in soft LungMA hydrogels possessing an average total cell of approximately 0.05 mm^2^, and soft GelMA hydrogels promoting an average total cell area of 0.04 mm^2^.

The total area of embedded organoids was reduced with increasing DOX concentration. At intermediate doses, approximately 50% of the total cell coverage consisted of the spheroid core within a DOX concentration range of 0.1–1 µM for soft LungMA and 0.01–0.1 µM for soft GelMA (Figure 7d). In soft GelMA matrices, the average invasive cell area of PDOS organoids continued to decrease with increasing DOX concentration, until it constituted less than 20% of total cell area. In contrast, PDOS invasive areas stagnated at 60% of total cell area from 1 to 100 µM DOX in soft LungMA. The retention of invasive cells in DOX-treated soft LungMA suggests that the lung-based microenvironment can promote chemoresistance in distal and intermediate invasive cells, in addition to those in the spheroid core. In addition to reducing invasion area, DOX treatment also reduced organoids core size, with all spheroid conditions possessing areas ranging between 0.009–0.011 mm^2^ at 100 µM DOX treatment (Figure 7c). This suggests that at higher DOX concentrations, spheroid core growth is impeded through cytotoxic or cytostatic effects on the cells after drug exposure. Interestingly, there was a moderate correlation between metabolic activity and invasion area in PDOs embedded in soft matrices, but not stiff matrices, likely due to the relatively minimal invasive area in stiff matrix-embedded PDOs, therefore warranting further investigation of such relationships in future work (Supplementary Figure S4). Here, we’ve shown that using cell invasion assays, in addition to metabolic activity measurements, can provide novel insights into the behavior of embedded organoids. Despite appearing chemoresistant when assessed metabolically, DOX treated, embedded organoids still displayed characteristics of chemotherapeutic susceptibility in the form of invasive cell reduction with increasing DOX concentration (Figure 7).

Regarding future work, additional techniques for organoid assessment, such as core-invasion embedded organoid separation, as reported by Weiss et al., could be implemented (Weiss et al., 2023). In their study, they used a hole-punch to separate the organoid core from the surrounding cells, allowing region-specific analysis. The varied impact of ECM composition to drug response influences on cell-ECM invasion and survival, and these same survival pathways may be more activated in the cores of embedded organoids compared to matrix-free organoids. Using this core-invasion separation technique in the context of PDOS may allow identification of patient-specific, region-specific targets for both primary/solid tumor OS and metastatic/migratory OS. Furthermore, both core size and invasive cell area increased over time, indicating that newly proliferated cells either localized to the core, or invaded into the ECM (Figure 4). This technique could thus be useful in determining the driving factors behind cell migration or core-localization.

Furthermore, this study relied on DOX for the treatment of PDOS organoids; however, cisplatin and high-dose methotrexate are clinically used in combination with DOX. Therefore, future studies could determine chemoresistance to these chemotherapeutic agents, in addition to DOX. Additionally, OS is known for its radioresistance; however, there is still a minority of OS patients who respond to radiotherapy (20%) (Li et al., 2019). As a result of this high radioresistance rate, the inclusion of radiotherapy in the treatment regime is not recommended (Spałek et al., 2021; Schwarz et al., 2009). However, in the minority of cases where patients are susceptible to radiotherapy, it would be advantageous to identify such susceptibility, especially when treatment methods such as MAP are ineffective (Zhou et al., 2020). Previously, Charalampopoulou et al. (2026) demonstrated the utility of U2OS and SAOS-2 OS cell line spheroids *in vitro* for assessing radioresistance, by determining the effect of radiation type and concentration on spheroid size, metabolic activity and histological appearance post-treatment. Therefore, in future, this approach could also be applied to OS PDOs, to provide a more comprehensive *in vitro* assessment of multiple treatment pathways, aside from chemotherapy alone.

Additionally, this study possessed some limitations, which should be addressed in future experimentation. For example, the availability of OS patient tissue is a major constraint, as despite being the most malignant bone cancer, acquiring and successfully generating and propagating cells from patient resection tumor is difficult as compared to immortalized cell line culture. This led to the use and optimization of cells from only one patient donor in this study. Multiple donors, with distinct phenotypic variations, such as age, primary tumor location, and response to chemotherapy, could be used in future studies to assess inter-patient variability in chemoresistance and invasion. Regarding the chosen matrices for 3D cell culture, as seen in Figure 2, the lung dECM is comprised of a variety of proteins such as laminins, and proteoglycans, that could influence cellular invasion, and are absent in gelatin. However, no experiments were conducted to isolate the contribution of these proteins towards invasion and resistance. Therefore, future studies could investigate protein-specific effects on cell behavior in LungMA, such as the effect of laminin-neutralizing antibodies on embedded organoid chemoresistance and invasion (Ramadan et al., 2022). In terms of matrix stiffness, this study investigated 1 kPa and 4 kPa matrices, although OS can arise in environments within the range of MPa and GPa, therefore, future work could be aimed towards exploring how stiffness effects PDO behavior across greater stiffness ranges, reflective of both bone and lung microenvironments (Menshikh et al., 2024). Regarding the influence of stiffness and matrix composition on invasion, we previously touched upon the potential influence of collagen type VI and collagen type I in LungMA and GelMA, and how their respective characteristics may promote matrix-specific cell behaviors. Previous studies have shown that collagen type VI facilitates matrix invasion and cell survival through activation of pathways such as FAK/Src, P13-AKT, and MAPK, which may provide some insight into the results obtained in this study (Aumailley et al., 1989; Liu et al., 2024; Chen et al., 2013; Rühl et al., 1999; Wang and Pan, 2020; Voiles et al., 2014; Liu et al., 2024; Wishart et al., 2020). However, more work must be conducted specifically targeting such pathways in order to properly elucidate such mechanistic relationships. Furthermore, DOX was administered prior to the peak invasion window of days 7–14 (Figure 4d). Evaluation of sample resistance post-treatment within this window may yield different results, such as underestimation of the inhibitory effects of DOX, to those that we garnered through our relatively early invasion model.

Overall, our study shows that the effect of stiffness tuneable ECM composition and use of image analysis techniques for the characterization of embedded organoids offers unique, multi-dimensional insights into OS morphology, invasion and chemoresistance and has potential for improving patient clinical outcomes through *in vitro* pre-clinical assessment.

## 4 Conclusion

This study establishes a controllable 3D cell culture platform for investigating the behaviour and treatment response of PDOS organoids within defined ECM microenvironments. While ULA-generated, matrix-free PDOS organoids intrinsically recapitulated key tumour characteristics including compact architecture and increased chemoresistance, embedding within compositionally distinct and stiffness tunable LungMA and GelMA hydrogels revealed additional phenotypes driven by extracellular matrix derived biochemical and biomechanical signals. Lung-derived matrices, particularly under increased stiffness, promoted sustained invasive behaviour and elevated resistance to doxorubicin, supporting the concept that metastatic niche-like environments can reinforce aggressive tumour states.

Importantly, the observed divergence between metabolic viability and invasion-based responses highlights a critical limitation of conventional single metric drug screening approaches and underscores the necessity of multidimensional functional readouts in 3D cancer models. The image-based analytical framework presented here enables quantitative assessment of spatially resolved organoid behaviour and provides a broadly adaptable strategy for interrogating microenvironment regulated tumour heterogeneity. Although demonstrated here for osteosarcoma, this approach is readily transferable to other cancer types and offers opportunities for region specific therapeutic targeting and patient tailored evaluation of metastatic potential. Collectively, these findings position ECM-defined organoid systems as powerful next-generation platforms for mechanistic cancer biology and predictive preclinical drug evaluation.

## Data Availability

The original contributions presented in the study are included in the article/Supplementary Material, further inquiries can be directed to the corresponding authors.
