# Supplementary material for: Matrix type influences embedded patient-derived osteosarcoma organoid invasion and response to treatment
Source: Front Pharmacol. 2026 Jun 2;17:1831729. doi: 10.3389/fphar.2026.1831729 (PMC13269220; doi:10.3389/fphar.2026.1831729)
Supplement: Supplementary file 1 [file Supplementaryfile1.docx]

**Supporting Information**


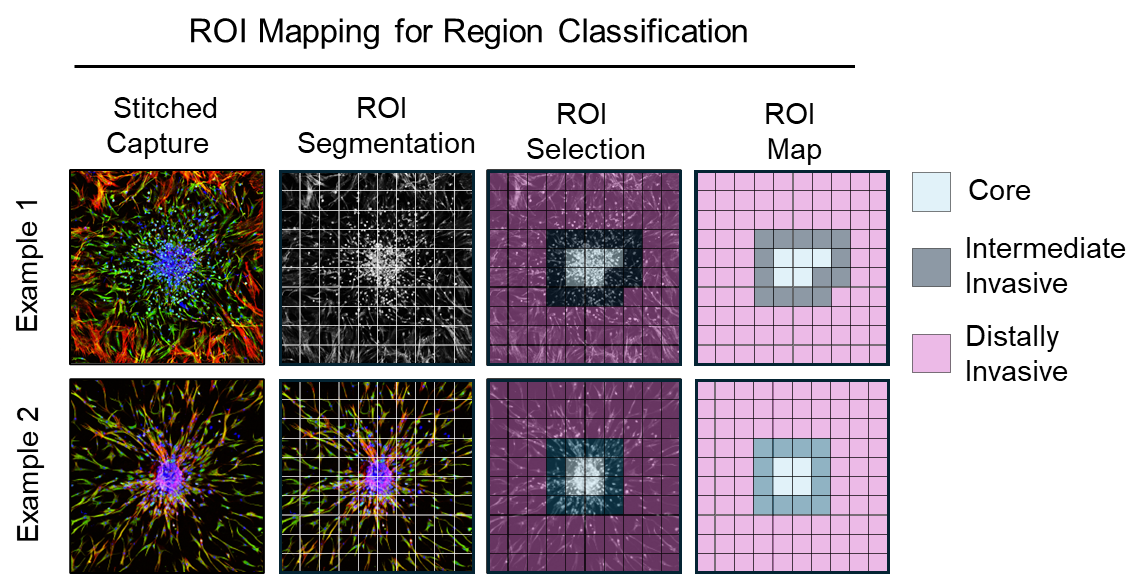


**Figure S1. Mapping of regions of interest (ROIs) for the classification of core and invasive areas in embedded organoids.** Stitched captures of embedded organoids were segmented into ROIs. The organoid cores were then identified based on their resemblance to non-embedded organoid-like appearance. Intermediate invasive cells were then determined as ROIs directly neighboring the core ROIs. Distally invasive cells were identified as all other cell-containing ROIs.


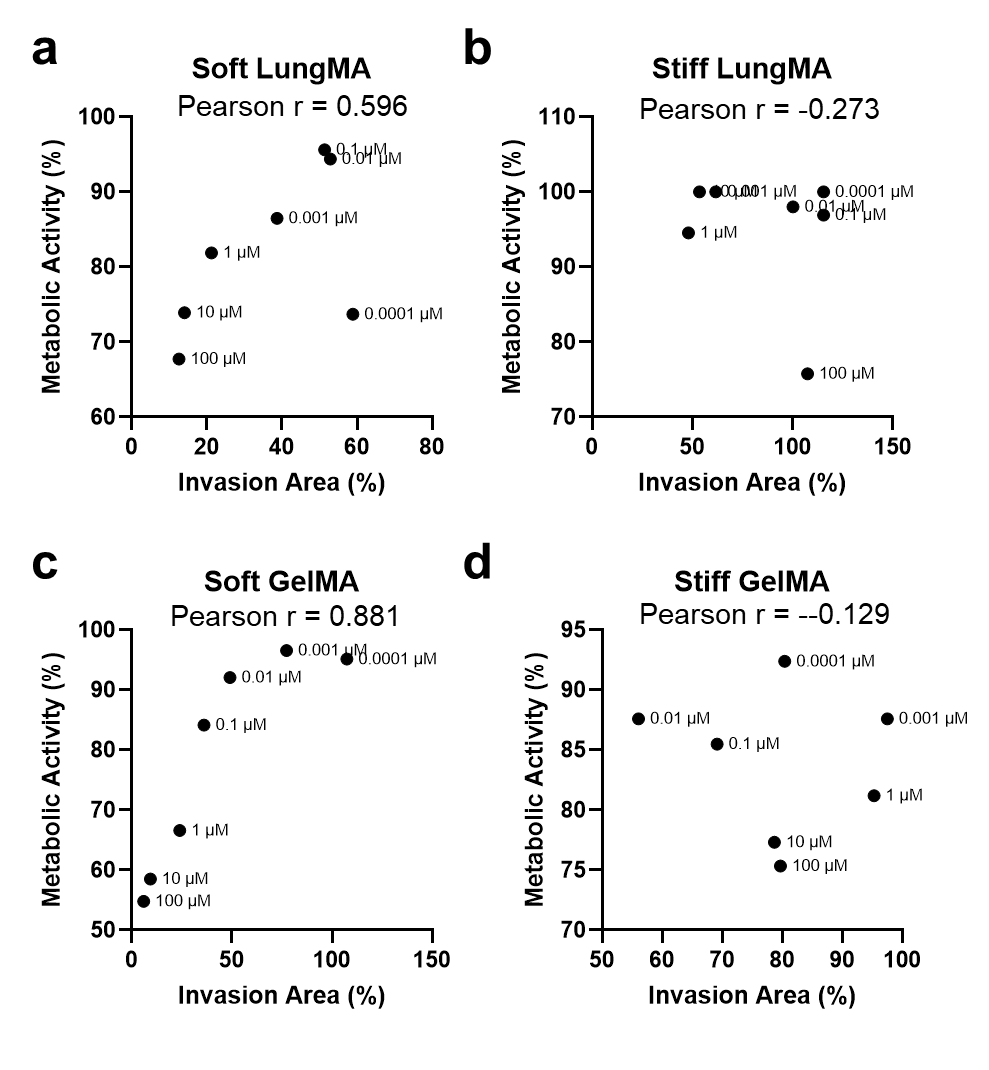


**Figure S2. Pearson coefficient between metabolic activity and invasion area of DOX treated embedded PDOs relative to vehicle controls. a)** Soft LungMA; **b)** Stiff LungMA; **c)** Soft GelMA; **d)** Stiff GelMA**.** The correlation coefficient (r) was calculated using the normalized metabolic activity and normalized invasive areas of embedded PDOs.

**Figure S3: ImageJ macro/script for standardized analysis of organoid and invasion areas:**

//Title: Analysis of Organoid Area and Invasion

//Note: Prior to using this macro, set the scale relevant for images undergoing analysis.

//1. Identify total organoid area (core + invasion)

run("Duplicate...", " ");

run("8-bit");

run("16 colors");

//2. Adjust brightness and contrast to help identify cells

waitForUser("Adjust Brightness/Contrast", "Adjust the b/c sliders in the b/c windown, then click OK here to continue the macro.");

waitForUser("Draw ROI", "Draw a freehand region encompassing total organoid area,\nthen click OK.");

run("Set Measurements...", "area perimeter shape feret's skewness kurtosis limit display add redirect=Clipboard decimal=3");

run("Measure");

//2. Analyze organoid core

run("Duplicate...", " ");

run("8-bit");

run("Brightness/Contrast...");

waitForUser("Adjust Brightness/Contrast", "Adjust the b/c sliders in the b/c windown, then click OK here to continue the macro.");

run("Threshold...");

//2.1 Adjust threshold

waitForUser("Adjust Threshold", "Adjust the threshold sliders in the 'Threshold' window, then click OK here to continue the macro.");

getThreshold(lower, upper);

setOption("BlackBackground", true);

run("Convert to Mask");

run("Fill Holes");

run("Fill Holes");

run("Despeckle");

run("Despeckle");

run("Remove Outliers...", "radius=25 threshold=98 which=Bright");

run("Remove Outliers...", "radius=25 threshold=5 which=Dark");

run("Set Measurements...", "area perimeter shape feret's skewness kurtosis limit display add redirect=Clipboard-1 decimal=3");

run("Analyze Particles...", "size=200-Infinity circularity=0.1-1.00 show=Outlines display exclude summarize overlay add");

**Figure S4: Imagej macro/script for standardized analysis of ECM-embedded organoid nuclei, actin and vimentin in fluorescence images:**

//Title: Analysis of Organoid Nuclei, Actin and Vimentin

//Notes: Prior to using this macro, set the scale relevant for images undergoing analysis. For this macro to work, you must copy and paste the image into ImageJ. Parameters such as size range in “Analyze Particles” may have to be altered to suit your image and channel specifications.

//1. Separate fluorescent channels

run("Duplicate...", " ");

run("Split Channels");

//2. Select fluorescent channel of interest (e.g. red for actin)

waitForUser("selectImage");

run("Duplicate...", " ");

run("8-bit");

//3. Adjust the brightness and contrast

run("Brightness/Contrast...");

waitForUser("Adjust Brightness/Contrast", "Adjust the b/c sliders in the b/c windown, then click OK here to continue the macro.");

//4. Adjust the threshold

run("Threshold...");

waitForUser("Adjust Threshold", "Adjust the threshold sliders in the 'Threshold' window, then click OK here to continue the macro.");

getThreshold(lower, upper);

//5. Complete analysis

setOption("BlackBackground", true);

run("Convert to Mask");

run("Set Measurements...", "area perimeter shape feret's skewness kurtosis limit display add redirect=Clipboard decimal=3");//setThreshold(0, 214);

run("Analyze Particles...", "size=20-Infinity circularity=0.0-1.00 show=Outlines display summarize overlay add");
